# The Use of Cisatracurium in Cardiac Surgery

**DOI:** 10.4274/TJAR.2025.241876

**Published:** 2025-12-22

**Authors:** Vikas C. Roy, Sakshi Mehta, Rajni Bala

**Affiliations:** 1 Global College of Pharmacy, Department of Pharmacy, Kahanpur, Punjab, India; 2 Adduct Healthcare Pvt. Ltd., Kharar, Punjab

**Keywords:** Cardiac surgery, cisatracurium, induction, neuromuscular blockers, residual paralysis

## Abstract

The introduction of neuromuscular blockers (NMBs) has revolutionized the practice of general anaesthesia, ushering in a new era where anaesthesia is conceptualized as a triad comprising narcosis, analgesia, and muscle relaxation. NMBs play a vital role in surgeries by facilitating tracheal intubation, preventing the movement of body and diaphragm, control of ventilation at normal partial pressure of carbon dioxide and counteraction of narcotic-induced truncal rigidity. However, the absence of specific guidelines for the selection and utilization of particular NMBs in various surgical contexts has led to inconsistencies within the healthcare system. Thus, a deep and thorough understanding of pharmacological aspects of NMBs is required for the selection and usage of particular NMB in clinical setting. Ideal NMBs are characterized by rapid onset, non-cumulative effects, independence from renal or hepatic function for elimination, rapid reversibility, and minimal adverse side effects. Among several NMBs, cisatracurium, an isomer of atracurium is a non-depolarizing intermediate-acting with characteristic features of high potency, smaller dosage requirement, no histamine release, no cardiovascular effects and elimination via organ-independent Hofmann reaction. Innumerable clinical experiments and trials suggest cisatracurium as safe, cost-effective, and better molecule with predictable recovery and no postoperative residual paralysis in comparison to other NMBs such as rocuronium, vecuronium, and pancuronium. In this review, we aimed to provide critical insights on the properties of NMBs first and then focused on the use of cisatracurium in cardiac surgeries.

**Main Points**

• Neuromuscular blockers (NMBs) evolved from natural agents to synthetic drugs to induce paralysis and muscle relaxation.

• An ideal NMB should have with characteristic features of high potency, smaller dosage requirement, no histamine release, no cardiovascular effects, and elimination via organ-independent Hofmann reaction.

• In cardiac surgery, cisatracurium is preferred for its minimal circulatory effects and faster recovery.

## Introduction

Neuromuscular blockers (NMBs) are the agents that induce paralysis and facilitate profound muscle relaxation and thereby, preventing muscle movement in clinical setting. Muscle relaxation was earlier maintained by deep inhalation of anaesthesia. The introduction and widespread usage of NMBs was a significant milestone for the development of balanced anaesthetic protocols.^1^ Historically, natives in South America used “curare”, a blocking agent for hunting and killing prey and later used it in specialized surgeries. NMBs were introduced in clinical practice for more than a decade now. From the initial use of naturally occurring tubocurarine, the evolution of NMBs has progressed towards the development of more potent synthetic benzylisoquinoline and amino-steroidal molecules with curare-like effects.^2^

Pharmacologically, NMBs exert their effects by blocking the neuromuscular transmission at the junction, resulting in paralysis of the affected muscle. NMBs may affect both pre- and postsynaptic neuromuscular junctions. Based on their action mechanism, NMBs are categorized as depolarizing and non-depolarizing agents.^3^ Depolarizing agent acts as agonist, binding at the acetylcholine receptive sites and causing an end-plate potential/depolarization of the muscle fiber. This process initiates two phases of depolarization block: phase I, characterized by muscle fasciculation (twitch), and phase II, or the desensitizing phase, during which the muscle becomes unresponsive to acetylcholinesterase, ultimately leading to neuromuscular blockade. In contrast, non-depolarizing agents act as antagonists, blocking receptor sites and preventing depolarization and neuronal transmission to the muscle.^4^

NMBs are also characterized on the basis of duration of action i.e., short-acting (succinylcholine), intermediate-acting (atracurium, cisatracurium, vecuronium and rocuronium) and long-acting (pancuronium) agents. Innumerable blocking agents with their individualistic features and chemical structures significantly influences factors such as blockage duration, recovery time, and the risk of postoperative residual paralysis in clinical settings.^5^ There are several other factors such as abnormal hepatic and renal functions, histamine release and malignant hypothermia that affect the potential and action mechanism of a certain NMBs. Thus, a better understanding of NMBs with effective blockade potential is the need of an hour. However, there are no specific guidelines or consensus data in the literature that could emphasize on the choice of preferrable NMB in a specialized surgery which is highly controversial among clinicians and surgeons.^6^ Therefore, this review aimed to provide insights on the different types of NMBs, their characteristic features, and the role of cisatracurium in various cardiac surgeries. By synthesizing existing knowledge and addressing gaps in understanding, this review seeks to offer valuable guidance to healthcare professionals grappling with the complex decision-making process surrounding NMB selection in specialized surgical contexts.

### Existing NMBs and Their Pharmacokinetics

The currently available agents are as follow:

**Succinylcholine:** Succinylcholine is the only depolarizing agent which is currently in use due to its favorable pharmacokinetic profile i.e., rapid onset and short duration of action. Succinylcholine triggers malignant hyperthermia and hyperkalemic response with a transient increase of 0.5-1.0 mEq L in plasma potassium levels.^7^

**Pancuronium:** Pancuronium was the first steroidal agent that had curare-like effects. It was isolated from the bark of Malouetiabequaertiana. Pancuronium has the ability to block the cardiac muscarinic receptors resulting in tachycardia. Therefore, the usage of pancuronium is discouraged due to slow onset, cardiac effects and prolonged action.^8^

**Vecuronium:** Vecuronium, an intermediate-acting blocker with stable hemodynamic profile, is metabolized into 3-desacetyl-vecuronium. However, it is not recommended in patients with hepatic or renal dysfunction as it is metabolized in the liver and excreted in bile and urine. The elimination half-life is approximately 45 to 60 minutes.^9^

**Rocuronium:** Rocuronium, a deacetoxy analogue of vecuronium has shorter onset but an intermediate duration of action depending upon the hepatic or renal functioning. Although rocuronium is not associated with histamine release, it has a little impact on hemodynamic profile and can cause allergic reactions.^10^ The half-life of rocuronium elimination is approximately 1-2 hours.

**Atracurium:** Atracurium is a mixture of ten isomers with intermediate blocking activity. Atracurium is an attractive option to use in patients with renal and/or hepatic dysfunction. However, intubating doses of atracurium (0.5 mg kg^-1^ or 2×ED_95_) can cause histamine release, tachycardia, and hypotension. The half-life of atracurium elimination is approximately 20 to 25 minutes.^11^

**Cisatracurium:** Cisatracurium, a stereoisomer of atracurium is an intermediate-acting non-depolarizing agent. It constitutes 15% of atracurium. Cisatracurium acts as a competitive antagonist to acetylcholine and binds to the nicotinic cholinergic receptor at the muscular junction. It effectively blocks the motor end-plate potential and inducing paralysis by disrupting the required conformational changes for ion channel opening. Clinically, it is found beneficial because of its duration of action and rate of spontaneous recovery, making it clinically beneficial in anaesthesia management. Notably, it is considered as a “cleaner” molecule with characteristic features of five-fold potency in comparison to atracurium, no histamine release, a smaller dosage requirement for tracheal intubation (0.1 mg kg^-1^ or 2×ED_95_) and elimination via Hofmann method which is independent of renal/hepatic functioning. The metabolization of cisatracurium leads to the formation of mono-quaternary acrylate metabolite and laudanosine. The role of laudanosine metabolite is controversial. Early studies in animal models suggested that laudanosine could induce seizure-like activity at high doses, such adverse effects have not been reported in humans.^12^ Importantly, the concentration of laudanosine was significantly lower in cisatracurium infused-surgical patients as compared to atracurium infused-surgical patients. This finding underscores the safety profile of cisatracurium, making it a preferable choice for long-term use in the intensive care unit (ICU) without the concerns associated with adverse effects linked to laudanosine accumulation. Approximately 77% of the drug undergoes degradation reaction and 15% is excreted unchanged in the urine. Its elimination half-life is approximately 20 to 25 minutes.^13^ Overall, cisatracurium emerges as a valuable asset in anaesthesia practice, offering effective muscle relaxation with a favorable safety profile and lower risk of laudanosine-related complications compared to other neuromuscular blocking agents.

### Ideal NMB as a Drug of Choice

Table 1 shows the comparative analysis of various NMBs in terms of potency, chemical structure variability and pharmacodynamic features, which is as follows:

**Chemical structure variability:** Most of the NMBs are quaternary ammonium compounds which are structurally similar to acetylcholine. Among them, succinylcholine, pancuronium, atracurium and cisatracurium contain bis-quaternary amines that make them more potent than mono-quaternary NMBs (rocuronium and vecuronium).^14^

**Potency:** Potency of the drug depends on the potential of the drug to block neuromuscular functions. Potency is inversely proportional to the dose of the drug and onset of time. If the potency of the drug is low, a larger dose is administered which may accelerate onset of action.^15^ The ascending order of potency of NMBs initiate from rocuronium with lowest potency followed by atracurium, vecuronium, cisatracurium and pancuronium with highest potency. Rocuronium has four to five times low potency than that of cisatracurium.^10^ In a study, Diaz et al.^14^ compared the relative potency, onset, duration of action, and reversal characteristics of cisatracurium with pancuronium in rabbits. The authors observed that the clinical time point for reversal and initial recovery was faster in cisatracurium irrespective of same onset.

**Pharmacodynamic parameters:** Relative efficacy of the agents is measured quantitatively as the ED_95_. ED_95_ is the average dose needed to produce 95% suppression of the adductor pollicis twitch response to ulnar stimulation. Hemmerling et al.^16^ provided an update on the usage of NMBs in cardiac surgery. The authors reported the comparative pharmacodynamic parameters (dose of intubation, onset time, ED_50_, ED_95_, elimination, clearance, hepatic/renal failure and histamine release) of widely used NMBs such as cisatracurium, pancuronium, vecuronium and rocuronium. In a study, Jirasiritham et al.^17^ compared the effectiveness and characteristics of atracurium and cisatracurium with respect to onset, duration of blockade, intubating conditions and hemodynamic parameters in 150 patients undergoing elective surgeries under general anaesthesia and found cisatracurium as a better NMB.

**Cardiac effects/Histamine release:** The major adverse events associated with the usage of NMBs include cardiac effects, histamine release and actions at extra-neuromuscular junction of cholinergic receptors. The older agents i.e., curare and succinylcholine and currently available pancuronium and atracurium are found to be causing such adverse effects.^18^ However, cisatracurium is a major attraction that shows no such signs of histamine release.

**Elimination:** The elimination pathway for NMB should be independent from hepatic or renal dysfunction. The metabolization, elimination and prolonged blocking effects of various agents such as pancuronium, vecuronium and rocuronium is dependent on the functioning of kidney and liver. However, atracurium and cisatracurium are considered best in case of multiple-organ failure as their mechanism is based on Hofmann’s elimination.^12^

**Haemodynamic stability:** In case of cardiac surgeries, haemodynamic stability is a must. In a study, Hemmerling et al.^16^ have mentioned that cisatracurium has no effect on haemodynamic factors including blood pressure, heart rate and cardiac output whereas other NMBs such as pancuronium, vecuronium and rocuronium have a significant effect on these parameters. Significant hemodynamic changes such as hypotension, tachycardia, and bronchospasm were also evident with the use of various prescribed agents. Particularly, vecuronium and atracurium administration are associated with hypotension and flushing. The use of cisatracurium is preferrable in case of bradycardia and unstable hemodynamic parameters.^11^

**Induction and maintenance:** Good intubating condition and hemodynamic stability are based on induction. Rocuronium and cisatracurium gives good intubating condition. During maintenance of surgery, muscle relaxation is necessary due to hypothermia, shivering, defibrillation.^19^ In addition, malignant hyperthermia is a rare genetic disorder that has shown to be triggered by NMBs administration along with halogenated hydrocarbons anaesthetics. It causes an excessive release of calcium from the sarcoplasmic reticulum of skeletal muscle. The early features include tachycardia, cyanosis, and muscle rigidity and the mortality rate is 80%, if left.^20^

**Postoperative residual paralysis:** Postoperative residual paralysis is a matter of concern and a great hurdle in cardiac surgery. It is most likely to happen with long-acting NMBs like pancuronium but very less frequent with intermediate-acting NMBs such as cisatracurium.^21^

### NMBs in Cardiac Surgeries

The muscle relaxants play an active role in cardiac surgery by facilitating tracheal intubation, preventing pulmonary movement, ventilation control by the partial pressure of arterial carbon dioxide, counteracting narcotic-induced truncal rigidity and prevention of shivering during hypothermic bypass. The choice of muscle relaxant for patients undergoing cardiac surgery may be influenced by the circulatory effects evoked by these agents.^22^ The circulatory effects produced by muscle relaxants include release of histamine, autonomic ganglionic block, blockage of cardiac muscarinic effects (vagolytic), noradrenaline release, potassium ion kinetics and drug interaction. The NMB with minimal circulatory effects is desirable in cardiac surgeries. The role of muscle relaxants in cardiac surgery is defined in three phases:

**Induction of anaesthesia:** Endotracheal airway is inserted using NMB that provides good to excellent intubating conditions in the patients undergoing cardiac surgery. The prime purpose of induction is to provide hemodynamic stability. Generally, rocuronium is used in cardiac surgeries due to early intubation as compared to other NMBs.^23^

**Maintenance of NMB during surgery:** NMB dosing is titrated to achieve blockade of the core muscles and the blockade is maintained during the surgery to avoid any inadvertent movement of the patient, hypothermia, shivering and defibrillation.^24^ Hypothermia alters the distribution and metabolism of NMBs, influences twitch response and increases duration of action of NMB with the reduction of 2 °C in body temperature. Bolus and continuous infusion are two discrete methods of NMB administration. Bolus mode of NMB administration provide sufficient paralysis whereas continuous infusion may cause postoperative residual paralysis. Postoperative residual paralysis is most common with long-acting NMB, pancuronium whereas less frequent with intermediate-acting NMB, cisatracurium. In a study, Van Oldenbeek et al.^25^ reported considerable degree of residual block [median: train-of-four (TOF) = 0.23] in 13 of 20 patients with modest dose of pancuronium (median = 0.11 mg kg^-1^ total) after cardiac surgery. In another study, Baillard et al.^26^ reported postoperative residual paralysis with vecuronium in 33% of patients undergoing different types of surgery (TOF ratio <0.7). A large bolus of cisatracurium (8X ED_95_) administration is recommended for initial blockade in peri-operative period and small bolus dose can be used during the surgery, if required to avoid postoperative residual paralysis.^27^

**Post-operative period:** Recovery from the blockade is a must in post-operative period. Any TOF response of less than 0.9 should be reversed. Though muscle relaxants are not needed post-operatively in stable patients or in case of fast-track surgeries however, if required, cisatracurium is preferred to avoid bradycardia.^28^ In a study, Cammu et al.^29^ compared the recovery time after continuous infusion of cisatracurium and rocuronium and observed the time interval of 10+9 min for cisatracurium and 18+13 min for rocuronium between end of infusion and reappearance of TOF ratio of 0.9. In some cases, reversal agent such as sugammadex (2 mg kg^-1^ of dose), is also used which is carefully titrated to avoid bradycardia or tachycardia.^29^ The following cardiac surgeries are taken into consideration:

**Coronary artery disease:** Coronary perfusion maintenance and less utilization of myocardium are the pre-requisite goals for the management of patients with coronary artery disease. These factors can be achieved by controlling heart rate and maintaining demand-supply ratio. In this regard, a muscle relaxant providing good intubating condition, hemodynamic stability, prevention from hypothermia and rapid recovery is selected for the patient undergoing cardiac surgery. NMBs with benign circulatory effects include vecuronium, rocuronium and pipecuronium.^30^ Atracurium and pancuronium have modest blood pressure and heart rate effects however, pancuronium is extensively being used for many years in patients with coronary artery disease. Pancuronium is useful in offsetting negative inotropic and chronotropic effects of anaesthetic drugs especially in opioid-induced bradycardia. Myocardial ischemia is most likely to occur during tracheal intubation irrespective of NMB choice. In a study, 12 patients with coronary artery disease were administered pancuronium and reported myocardial ischemia in 3 patients due to associated increase in heart rate. Hence, the choice of NMB is dependent on basal heart rate and its maintenance.^31^ A paradigm shift from traditional surgical procedures to fast-track concept highlighted the role of NMBs in cardiac surgeries and demarked the usage of opioid and benzodiazepines. In this regard, NMB agents associated with early recovery, early tracheal extubation and least complications should be used. In a study, Murphy et al.^22^ showed a significant delay in tracheal extubation with pancuronium (median, 500 min; range: 240-1305 min) as compared to rocuronium (median, 350 min; range: 210-1140 min). Hence, the selection of NMB relies on the associated circulatory effects and recovery profile.

**Valvular heart disease:** The patients with mitral stenosis, aortic stenosis, combination of aortic stenosis and mitral stenosis, and other combinations have compromised cardiac filling and contractility.^32^ Maintenance of adequate stroke volume is dependent on heart rate and systemic blood pressure in patients with aortic stenosis. Pancuronium promotes forward left ventricular stroke volume and increase cardiac impulse transmission through the atrioventricular node. Short-acting and intermediate-acting muscle relaxants such as rocuronium or cisatracurium are often the logical choices in patients with aortic and mitral stenosis as these agents are associated with minimal changes in heart rate and systemic blood pressure and forward flow maintenance. Further, pancuronium should be preferred in patients with aortic incompetence, mitral incompetence and atrial fibrillation (AF) whereas rocuronium and vecuronium should be used in patients with stenosis to decrease the heart rate. Pancuronium should be avoided in patients with mitral stenosis.^33^

**Cardiomyopathies:** Cardiomyopathy occurs due to muscle weakness and rigidity. It includes hypertrophic cardiomyopathy, dilated cardiomyopathy, restrictive cardiomyopathy and arrhythmogenic ventricular dysplasia. The treatment protocols include implantable cardioverter defibrillator, ventricular assisted device and cardiac revascularization. The cardiac revascularization practice includes blockage of respiratory movement using NMB for planned intubation and mechanical ventilation.^34^ However, prolonged postoperative ventilation is no longer necessary or desirable. The major goal in cardiomyopathy is to minimize negative inotropic effect, maintain preload, prevent increase in afterload causing vasodilation and avoid tachycardia. Pancuronium is associated with increased heart rate and delayed extubation, vecuronium and rocuronium produce positive inotropic effects and vecuronium shortens refractoriness. Based on these facts, vecuronium, rocuronium or cisatracurium should be preferred in patients with myopathy impairment or hypertrophic cardiomyopathy to avoid bradycardia and vasodilation.^35^ Pancuronium or cisatracurium should be preferred in patients with restrictive cardiomyopathy to avoid tachycardia and vasodilation. Rocuronium or vecuronium is preferred in patients with no automated implantable cardioverter defibrillator. Cisatracurium is preferred in all types of cardiomyopathies to avoid bradycardia and vasodilatation.^36^

**Minimally invasive cardiac surgery:** Minimally invasive cardiac surgery (MICS) comprises of minimally invasive direct coronary artery bypass (MIDCAB), robotic-assisted cardiac surgery, AF ablation surgery, and minimally invasive approaches to the mitral valve, left and right atria, and minimal-access aortic valve surgery.^37^ The procedure include the necessity of one-lung ventilation, use of cerebral oximetry, additional large-bore jugular vascular access, and advanced skills in transesophageal echocardiography. Several studies highlighted c the benefits of conventional coronary artery bypass grafting and MIDCAB.^38, 39^ The major benefits of MICS include reduced postoperative pain, shorter hospital stay, better cosmesis, and quicker resumption of normal activities, which form the basis of selection of NMB. Thus, the preferred properties of NMB include no cardiovascular or hemodynamic adverse effects, speedy recovery, highly potent, less inactive metabolites and unaffected by renal/hepatic impairment.

**Heart, lung and heart-lung transplant:** The heart transplantation procedure preserves the donor sinus node function. Sympathetic and parasympathetic reinnervation is established to improve heart rate and contractile response and to avoid bradycardia. The patients undergoing heart, lung and heart-lung transplantation require hemodynamic stability and post-operative ventilation.^40^

**Cardiac patients with renal or hepatic dysfunction:** Hepato-biliary disorders are associated with delayed onset of action, delayed metabolism, resistance to drugs. The course of action depends on NMB dosing where repeated dosing causes prolongation of action. Most of the NMBs such as pancuronium, vecuronium, rocuronium are metabolized through bile/liver and excreted through kidney which leads to increase elimination half-life. In diseased condition like cirrhosis, there is increase in volume of distribution, slow onset and prolong duration of action.^41^ NMBs like atracurium and cisatracurium have liver and renal-independent elimination. To some extent, vecuronium has renal-independent elimination. Laudanosine is the metabolite of atracurium and cisatracurium. In a clinical study, Zhou et al.^13^ suggested the use of atracurium/cisatracurium in patients with renal and hepatic failure.

**Cardiac intensive care:** NMBs are regularly used in ICUs to facilitate mechanical ventilation, eliminate patient-ventilator dyssynchrony, reducing intra-abdominal pressure and gas exchange by improving chest wall compliance. However, prolonged blockade is associated with ICU-acquired weakness. Succinylcholine is preferred for rapid sequence intubation. In patients with open chest surgery, preferred NMBs are vecuronium, atracurium and cisatracurium.^42^

Innumerable studies in the literature also recommended cisatracurium in cardiac surgeries which are listed in Table 2.

### Residual Paralysis and Reversal Agents

Muscle relaxation is one of the major elements of the general anaesthesia triad along with the usage of hypnosis and analgesia. However, the reversal procedures at the end of surgery are the matter of concern. Several significant clinical issues including perioperative management of neuromuscular blockade, adequate monitoring and residual neuromuscular blockade have been identified. Because of these concerns, long-acting NMBs are rarely used in the clinical practice. An ideal neuromuscular blocking agent should not only provide profound relaxation but also offer ease of reversal. Thus, ensuring the safety of neuromuscular blockade usage necessitates the availability of agents that can be quickly and reliably reversed.^5^ Reversal agents are of great importance in order to restore neuromuscular function in patients and to reduce the risk of postoperative residual paralysis. Several reversal agents hold clinical relevance offering distinct advantages and considerations, which are as follow:

**Acetylcholinesterase inhibitors:** These inhibitors function by antagonizing the action of NMBs through a mechanism involving the enzyme acetylcholinesterase. Normally, acetylcholinesterase functions to degrade acetylcholine at the neuromuscular junction but acetylcholine accumulates upon inhibition, competing with NMBs for receptive sites on nicotinic receptors.^47^This competition facilitates the reversal of neuromuscular blockade. The median recovery time following administration of acetylcholinesterase inhibitors is typically around 15 minutes, although this timeframe may vary depending on individual patient factors and clinical circumstances. It is imperative to recognize that the increased concentration of acetylcholine resulting from these inhibitors can also affect muscarinic receptors, potentially leading to adverse effects such as bradycardia and bronchoconstriction. To mitigate these risks, the concurrent administration of an antimuscarinic drug like glycopyrrolate is recommended. This adjunctive therapy helps to counteract the muscarinic effects of acetylcholine accumulation, thereby minimizing the occurrence of unwanted side effects and ensuring a smoother recovery from neuromuscular blockade reversal.^48^

**Sugammadex:** Sugammadex a crucial reversal agent in anaesthesia practice is comprised of a gamma-cyclodextrin compound. It facilitates the encapsulation of NMBs within the plasma. This process results in the formation of a tightly bound, inactive sugammadex-aminosteroidal complex, which is subsequently excreted via the urine.^49^ Sugammadex has a remarkable ability to rapidly reverse even deep or profound levels of neuromuscular blockade, swiftly restoring normal muscle function. However, it is important to exercise caution, as its use is contraindicated in patients with a creatinine clearance of less than 30 mL min^-1^.^50^ Furthermore, while hypersensitivity reactions to sugammadex are rare in clinical settings, they remain a potential concern that necessitates vigilance. Despite these considerations, sugammadex remains an invaluable tool in the management of neuromuscular blockade, offering a swift and effective means of reversing its effects.

## Conclusion

In conclusion, NMBs serve as valuable adjunctive therapy across a spectrum of surgical procedures, including transplantations and cardiac surgeries. However, the optimal utilization of a specific NMB necessitates a comprehensive understanding of its intricate properties. Over recent decades, significant strides and breakthroughs have reshaped the landscape of cardiac anaesthesia and surgery. Despite historical preferences, such as the use of pancuronium in cardiac surgery, it has become evident that its pharmacodynamics and pharmacokinetics render it less than ideal. In contrast, cisatracurium emerges as a superior choice for NMB due to its profound properties, highlighting the importance of staying abreast of advancements in pharmacology to optimize patient outcomes and ensure the efficacy and safety of anaesthesia protocols.

## Figures and Tables

**Table 1. Comparative Features of Various NMBs:** 

**NMBs**	**Action time**	**Onset of action (min)**	**Duration of action (min)**	**Chemical structure variability**	**Adverse effects/features**
Succinylcholine	Short-acting	1	10	Single chemical entity	Malignant hyperthermia, masseter muscle rigidity, bradycardia
-	Intermediate-acting	2	43	Mixture of 10 isomers	Release of histamine, toxic metabolite called laudanosine accumulation in individuals with renal failure
Cisatracurium	Intermediate-acting	2-3	45	Stereoisomer of atracurium	Does not cause release of histamine
Vecuronium	Intermediate-acting	3	33	-	May cause prolonged paralysis and promote muscarinic block
Rocuronium	Intermediate-acting	1-2	33	Deacetoxy analogue of vecuronium	May promote muscarinic block
Pancuronium	Long-acting	3-4	75	Steroidal compound	Tachycardia

NMBs, neuromuscular blockers; min, minutes.

**Table 2. Various Studies in Favour of Cisatracurium Administration in Cardiac Surgeries:** 

**Study type**	**Method**	**Opinion**	**References**
Randomized clinical trial	One hundred patients were randomized to receive succinylcholine or cisatracurium	A strategy of using cisatracurium resulted in better operating conditions	^43^
Prospective study	Eighty-seven patients were administered with cisatracurium or atracurium	Atracurium or cisatracurium administered in a single dose to facilitate endotracheal intubation does not result in residual postoperative paralysis	^44^
Prospective study	Twenty adult patients undergoing cardiac surgery received pancuronium	Residual paralysis with Pancuronium was observed. It should be replaced with rocuronium, atracurium or cisatracurium	^25^
Retrospective study	Seven thousand and one hundred thirteen patients were analyzed in which 3,751 received pancuronium and 3,362 received rocuronium	Both rocuronium and cisatracurium are now considered extremely attractive agents	^45^
Prospective study	Compared recovery time after continuous infusion of cisatracurium and rocuronium	Cisatracurim should be preferred over rocuronium	^46^
Review	-	Cisatracurium or rocuronium is recommended for neuromuscular blockade in modern cardiac surgery	^16^
